# One size does not fit all: understanding individual living kidney donor risk

**DOI:** 10.1007/s00467-019-04456-8

**Published:** 2020-01-02

**Authors:** Elham Asgari, Rachel M. Hilton

**Affiliations:** Guy’s and Saint Thomas’ NHS Foundation Trust, London, UK

**Keywords:** Kidney transplant, Donor risk, Long-term outcomes, End-stage kidney disease, Morbidity, APOL1, Shared decision making

## Abstract

Living donor kidney transplantation is the optimal treatment for end-stage kidney disease (ESKD) but confers a risk upon the donor, both in the short term and many years after donation. While perioperative mortality is low and longevity does not appear to be adversely affected, there are small increases in the risk of other important morbidities. The overall risk of ESKD among donors is low but appears to be three- to five-fold higher than among healthy non-donors, and this relative risk is even higher among donors of African ancestry. For these individuals, apolipoprotein L1 genotyping may be helpful. Kidney donors also have an increased risk of developing hypertension post-donation and a modestly increased risk of developing gout. Living kidney donation also increases the risk of gestational hypertension and preeclampsia while not affecting other important pregnancy outcomes. As our understanding of donor risk grows, it is important to counsel prospective donors according to their individual risk and so obtain better informed donor consent. As knowledge advances, it is also important that all clinicians who manage kidney transplant candidates have an up to date understanding of donor risk to inform shared decision making.

## What are the risks of kidney donation?

Living donor kidney transplantation is the best treatment for most people with end-stage kidney disease (ESKD), resulting in superior graft survival, reducing the size of the deceased donor kidney waiting list and facilitating early, even pre-emptive, transplantation [1]. Since the first living donor kidney transplant in 1954 [2], living kidney donation has expanded worldwide and now accounts for around a third of the UK kidney transplant program [3]. This has led to growing concern about donor risk, particularly in the longer term. This is difficult to predict, particularly in younger donors who have a long length of life remaining to them. Most published work reports short-term outcomes, which may or may not be appropriately extrapolated into the longer term. In this article, we present an up to date appraisal of the literature on the risks of kidney donation.

## Peri-operative complications

Lentine et al. studied peri-operative complications of living donor nephrectomy performed between 2008 and 2012 by linking the US transplant registry with records from a large consortium of hospitals [4]. Within a cohort of close to 15,000 donor nephrectomies, they observed perioperative complications in 16.8%, most commonly gastrointestinal (4.4%), followed by bleeding (3%), respiratory (2.5%) and surgical/anaesthesia-related injuries (2.4%). Only 2.5% of donors had a major (Clavien grade IV or higher) complication. Interestingly, in comparison with white donors, black donors had a 26% increased risk of having any complication and a 56% increased risk of major complication, after adjustment for demographic, clinical, procedure and centre factors. Obesity, haematological disorders, psychiatric conditions and robotic nephrectomy were also associated with a higher risk of major complications. High volume centres (> 50 donor nephrectomies per annum) reported a lower risk of complications.

Kortram et al. undertook a meta-analysis of short-term complications of minimally invasive donor nephrectomy [5]. The overall risk of an intraoperative complication was 2.3%, mainly bleeding (1.5%). The rate of post-operative complication was 7.3%, including infectious complications (2.6%) and bleeding (1%). Conversion to open nephrectomy was required in only 1.1% of cases.

## Short- and long-term mortality

Death following donor nephrectomy is generally agreed to be rare, although most studies report a relatively short-term follow-up. For example, in the meta-analysis undertaken by Kortram et al. [5], the 30-day mortality rate was 0.01%. In a US registry study of over 80,000 live donor nephrectomies carried out between 1994 and 2009, Segev et al. observed 25 deaths within 90 days, giving a surgical mortality of 3.1 in 10,000 [6]. This is lower than the reported mortality for comparable surgical procedures, such as laparoscopic cholecystectomy (18 per 10,000 cases) [7] and non-donor nephrectomy (260 per 10,000 cases) [8]. This was one of the first studies to assess longer-term post-donation mortality. After a median follow-up of 6.3 years, there was no significant difference in mortality between the donor population and over 9000 age- and comorbidity-matched controls.

A large Canadian study assessed the risk of death among 2028 kidney donors who donated between 1992 and 2009 compared with 20,280 healthy matched controls [9]. After a median follow-up of 6.5 years, they observed a lower risk of death and major cardiovascular events in the donor group in comparison with the non-donors (2.8 vs. 4.1 events per 1000 person years; hazard ratio (HR) 0.66, 95% confidence interval (95%CI) 0.48–0.90). Other studies assessing longer-term post-donation mortality have likewise reported a similar or lower risk of death in comparison with controls. For example, among 481 Japanese donors who donated a kidney between 1970 and 2006, the survival rate at up to 30 years was superior to survival in an age- and gender-matched cohort from the general population [10].

However, in a study of 1901 Norwegian donors who donated a kidney between 1963 and 2007 with a median follow-up of 15.1 years, in comparison with 32,621 controls who were potentially eligible to be kidney donors and followed up for 24.9 years, the risk of all-cause mortality was similar in the first decade after donation but increased in 25 years after donation by which time the donor group had a cumulative all-cause mortality of 18% compared with 13% in the non-donor group (adjusted hazard ratio (aHR) 1.3, 95% CI 1.1–1.5, *p* < 0.001) [11]. This was perhaps because the controls were selected from a rural county where life expectancy exceeds that of the general Norwegian population, whereas the donor population was more diverse. Reassuringly, in a recent systematic review and meta-analysis of 52 studies comprising 118,426 living kidney donors and 117,656 non-donor controls, with average follow-up between 1 and 24 years, 6 studies reported on all-cause mortality and found no difference between donors and controls [12].

## End-stage kidney disease

It is perhaps intuitive to imagine that giving up 50% of kidney function might lead to an increased risk of kidney failure in kidney donors. Many studies have attempted to estimate the post-donation risk of ESKD, but to do so accurately is challenging as it requires not only long-term follow-up of donors but also identification of an appropriate control population. Many studies have compared the risk of ESKD in kidney donors with that of the general population, which is unfair, as proceeding kidney donors are generally healthier than the general population with fewer risk factors for developing kidney disease.

In the Norwegian study previously mentioned [11], the incidence of ESKD among donors was 302 per million person-years in comparison with 100 per million person-years in the controls. The hazard ratio for ESKD in the donor group was 11.38 (4.37–29.63, *p* < 0.001). This study prompted much discussion, particularly regarding its applicability to other populations. Importantly, of the nine donors who developed ESKD, all were blood relatives of their recipients and causes of kidney failure were mostly immunological, possibly reflecting a genetic predisposition. Furthermore, the control group came from a single rural county whereas the donors came from all over Norway. Hence, there was potential bias in environmental factors affecting the donors in comparison with the controls.

A US study compared the risk of ESKD among 96,217 kidney donors between 1994 and 2011 with a median follow-up of 7.6 years, with a control population of 20,024 individuals with follow-up of 15 years [13]. In total, 99 donors developed ESKD in comparison with 36 controls equating to a 15-year estimated risk of ESKD of 30.8 per 10,000 population in the donors and 3.9 per 10,000 in the controls (*p* < 0.001). This difference was observed in both black and white individuals. Although the risk of ESKD was increased in the donor group, the overall risk was low; the estimated lifetime risk of ESKD was 90 per 10,000 donors, 326 per 10,000 unscreened non-donors from the general population and 14 per 10,000 healthy non-donors. It is worth noting that the control group was drawn from individuals enrolled into a research survey between 1988 and 1994 whereas the study group had donated between 1994 and 2011. It is therefore possible that the higher incidence of ESKD in donors was in part due to increased recognition of kidney disease and more aggressive screening for conditions such as high blood pressure and diabetes during this later time period. Nevertheless, potential kidney donors are and always have been highly screened and are unlikely to harbour significant risk factors for conditions that predispose to ESKD.

Recently, KDIGO in collaboration with the Chronic Kidney Disease Prognosis Consortium has conducted a meta-analysis of seven general population cohorts to estimate ESKD risk based on ten demographic and health characteristics [14]. They compared the 15-year projected ESKD risk in this cohort of 4,933,314 participants followed for a median of 4 to 16 years with the observed risk in 52,998 US living kidney donors. The factors that influenced ESKD risk included younger age, black race, male gender, lower glomerular filtration rate (GFR), smoking history and the presence of hypertension, albuminuria, obesity or diabetes. The highest risk of ESKD was in young black donors. On average, the 15-year risk of ESKD was 3.5 to 5.3 times higher in the donors compared with non-donors. Based on these data, an online risk assessment tool has been developed that can be used to estimate an individual’s risk of ESKD based on key baseline characteristics (http://www.transplantmodels.com/esrdrisk/). As this tool is based on US population data, it is not validated for other populations or racial groups, nor does it take into consideration the genetic donor-recipient relationship that can negatively impact upon donor risk [15]. Steiner argues that the tool may underestimate the risk of ESKD in younger donors, as important risk factors such as hypertension and diabetes have not yet developed; hence, it is difficult to accurately predict the long-term consequences of kidney donation for younger individuals [16].

## Hypertension and cardiovascular risk

Reduction in nephron mass is associated with increased blood pressure [17]. Considering that kidney donation is associated with a 50% reduction in kidney tissue, it is plausible to imagine that this could be associated with an increase in blood pressure. Several studies have compared the prevalence of hypertension in kidney donors in comparison with the general population. Among 3700 normotensive US kidney donors who donated between 1963 and 2014, followed up for a mean of 16.6 years, Sanchez et al. noted that over a quarter of the study population developed hypertension [18]. As in the general population, a higher risk of incident hypertension was associated with older age, family history of hypertension, higher BMI, higher fasting serum glucose, higher systolic and diastolic blood pressure, hyperlipidemia and being a smoker. Although the majority (90%) of this donor population were white, it was noted that white donors had a 30% lower risk of developing hypertension.

In a prospective study, 1214 normotensive Swiss kidney donors were followed for a mean period of 31.6 months to assess the 1- and 5-year occurrence of hypertension [19]. Using the Framingham risk calculator, the predicted risk of hypertension at 1-year post-kidney donation was increased by 3.64 (95% CI 3.52–3.76; *p* < 0.001). Interestingly, among those who remained normotensive 1 year post-donation, the risk of hypertension was no higher than in the healthy Framingham population. In all donors, the prevalence of microalbuminuria increased from 4.8 to 10.4% and hypertension was the main driver for this. Whether this increased risk of hypertension and/or microalbuminuria translates into renal dysfunction or other morbidities or mortality post-donation remains to be seen. In a different 3-year prospective follow-up of 182 kidney donors and 173 healthy controls, blood pressure was not different between the two groups at each follow-up visit (at 6, 12, 24 and 36 months) and 24-h blood pressure monitoring results were similar in 135 donors and 126 controls (mean (SD) systolic blood pressure, 120.7 ± 9.7 mmHg vs. 120.0 ± 11.2 (*p* = 0.6)) [20].

To assess whether the risk of hypertension is higher in black kidney donors, Doshi et al. [21] studied 103 African American donors who donated a kidney between 1993 and 2006 in comparison with 235 matched healthy controls from the Coronary Artery Risk Development in Young Adults (CARDIA) prospective cohort study. The follow-up period was 6.8 ± 2.3 years after donation and 6.4 ± 2.2 years after cohort entry. The donors were found to have an increased risk of hypertension (42/103 (40.8%) versus 42/235 (17.9%)), absolute risk difference 22.9% (95%CI 12.2–33.6%) and relative risk 2.4 (95%CI 1.7–3.4). Worryingly, more than half of the hypertensive donors were untreated for their hypertension, which highlights the importance of proper follow-up in this group.

To analyse the association between GFR and cardiovascular risk, Moody et al. [22] prospectively studied 124 living kidney donors and healthy controls looking at factors such as left ventricular mass (using cardiac MRI), left ventricular function and aortic stiffness. They found that 12 months post-donation, donors had a reduction in isotopic GFR of 30 ± 12 ml/min/1.73 m^2^ compared with controls. Donors also had a significant increase in left ventricular mass (+ 7 ± 10 vs. − 3 ± 8 g; *p* < 0.001) and reduced aortic distensibility (− 0.29 ± 1.38 vs. + 0.28 ± 0.79 × 10^−3^ mmHg^−1^; *p* = 0.03). Donors had greater risk of developing detectable highly sensitive troponin T (odds ratio, 16.2 (95%CI 2.6–100.1), *p* < 0.01) and microalbuminuria (odds ratio, 3.8 (95%CI, 1.1–12.8), *p* = 0.04). Serum uric acid, parathyroid hormone, fibroblast growth factor-23 (FGF-23) and high-sensitivity C-reactive protein all increased significantly. There were no changes in ambulatory blood pressure. These findings suggest that reduced GFR should be regarded as an independent causative cardiovascular risk factor. However, Garg et al. [9] compared the risk of cardiovascular events including myocardial infarction, coronary angioplasty, coronary bypass surgery, stroke, carotid endarterectomy, repair of abdominal aortic aneurysm or peripheral vascular bypass surgery in 2028 kidney donors in comparison with 20,280 matched non-donor controls followed for a median of 6.5 years (maximum 17.7 years). There was no difference in the rate of cardiovascular events between the two groups. These studies highlight the importance of longer-term donor follow-up to ensure optimal medical management.

## Gout

A decrease in GFR leads to reduced uric acid excretion, an increase in serum uric acid, and is a known risk factor for gout [23]. It is therefore plausible to predict an increased incidence of gout in living kidney donors. In a study of 201 kidney donors and 198 controls, Kasiske et al. [24] showed that kidney donors had a 28% lower GFR at 6 months (94.6 ± 15.1 (SD) vs. 67.6 ± 10.1 ml/min/1.73 m^2^; *p* < 0.001) and a 8.2% increase in uric acid level (4.9 ± 1.2 vs. 5.3 ± 1.1 mg/dl; *p* < 0.001).

In a retrospective study of 1988 Canadian living kidney donors who donated a kidney between 1992 and 2010, compared with 19,880 matched non-donor controls, with a median of 8.4-year follow-up (maximum 20.8 years), Lam et al. [25] found that donors were more likely than non-donors to be given a diagnosis of gout (3.5 vs. 2.1 events/1000 person-years; HR 1.6; 95%CI 1.2–2.1; *p* < 0.001) and were more likely to be given a prescription for allopurinol or colchicine (odds ratio 3.2, 95%CI 1.5–6.7, *p* = 0.002). There was, however, only a modest increase in the absolute incidence of gout among donors in comparison with controls (6.8% vs. 4.9% at 20 years post-donation).

Using a unique database that integrates national registry identifiers of US living kidney donors between 1987 and 2007 with billing claims from a private health insurer between 2000 and 2007, Lam et al. [26] identified the risk factors for gout in 4650 kidney donors, 13.1% of whom were African American. By 7 years, African Americans were almost twice as likely to develop gout as Caucasian donors (4.4% vs. 2.4%; aHR 1.8, 95%CI 1.0–3.2). Older age (aHR per year 1.05) and male gender (aHR 2.80) were also associated with an increased risk of gout. Male gender and African ancestry are known to be risk factors for gout in the general population [27, 28]. In this study, gout rates were similar in donors and age- and sex-matched general population non-donors.

## Metabolic bone disease

The reduction in GFR in kidney donors raises the question of a possible impact on bone metabolism in this population. Several studies report a rise in parathyroid hormone and FGF-23 levels and a reduction in serum phosphate following donation [22, 24, 29]. The ALTOLD study (Assessing Long Term Outcomes in Living Kidney Donors) [24] also noted a reduction in 1,25-dihydroxyvitamin D_3_ concentration in donors compared with normocalcaemic controls. It is not clear whether these changes in bone biomarkers have any long-term clinical consequence. In a retrospective cohort study to assess lower and upper-extremity fragility fractures, Garg et al. [9] reviewed the medical records of 2015 Canadian donors who donated between 1992 and 2009, in comparison with 20,150 healthy matched controls randomly selected from the general population. After a median follow-up of 6.6 years (maximum 17.7 years), they saw no difference in the rate of fragility fractures in donors compared with controls (16.4 vs. 18.7 events/10,000 person-years; rate ratio, 0.88; 95%CI, 0.58–1.32). Hence, there is no evidence of increased fragility fracture risk in living kidney donors.

## Psychological outcomes

Living kidney donation is a complex process that inevitably influences the psychosocial well-being of donors, and the expansion in living donor numbers has prompted investigation into this issue. The main aims of studies to date have been to identify potential risks to the psychological well-being of donors in order to facilitate better informed consent and to develop preventative measures. However, these avenues of enquiry are in their infancy and most studies to date have been single-centre, retrospective and with only short-term follow-up.

In a systematic review and meta-analysis investigating the course and predictors of physical and psychological health-related quality of life (HRQoL) post-donation, Wirken et al. [30] assessed 34 prospective studies published between 2002 and 2014, where at least one pre-donation and one post-donation assessment had been carried out. They found that shortly after donation, donors have a lower HRQoL, particularly in the physical domain, reflecting the effect of major surgery in a previously healthy individual. Between 3 and 12 months post-donation, the HRQoL was almost back to pre-donation levels, other than for fatigue, although this was similar to that observed in the general population. A limited number of studies have examined risk factors for impaired HRQoL, low psychological functioning before donation being the most consistent predictor.

The Renal and Lung Living Donors Evaluation study (RELIVE) evaluated the medical and HRQoL among kidney donors from three large US transplant centres [31], including 2455 kidney donors who donated between 1963 and 2005 with a mean follow-up of 17 years (range: 5–48 years). Over the long term, the majority of living kidney donors had an average or above average HRQoL. However, approximately 9% of donors had significantly reduced physical HRQoL, principally difficulty working or pain or other chronic conditions, and 9% had significantly impaired mental HRQoL, including symptoms of anxiety and depression. Obesity, history of psychiatric difficulty and non-white race were risk factors for reduced physical HRQoL (although 93% of the donors in this study were white); history of psychiatric difficulties was a risk factor for impaired mental HRQoL. Education, older donation age and being a first-degree relation to the recipient were protective factors. The fact that many transplant centres now accept donors with higher BMI makes this a noteworthy study. Enhanced pre-donation counselling and education, particularly weight loss counselling, and post-donation monitoring could improve outcomes for these donors.

When parents are assessed as kidney donors for their children it is important to consider the potential impact on family relationships. One Swedish study surveyed by questionnaire 76 donating and non-donating parents of kidney transplant recipients aged below 16 [32]. Interestingly, the non-donating parents reported more stress and anxiety than the donating parents. The donating parents reported improved relations with their recipients and better self-esteem after donation. There was no consistent change in relations between the donating parent and their partner, and relations between the donating parent and siblings of the recipient were unaffected. Most donors reported worse post-operative pain than expected and a third were dissatisfied with the level of peri-operative psychosocial support. A subgroup of the study population was interviewed a few years later and most continued to report positive psychosocial family dynamics and good relations with the recipient. However, several also reported psychological distress partly arising from unemployment as a result of the need to look after the child. This is important because living kidney donation is generally regarded as optimal treatment for children with ESKD; hence, parents may feel under an obligation to donate and some may find this stressful [33].

Two systematic reviews [34, 35] also found that most donors to paediatric recipients have a positive experience of donation. However, some studies raise the concern that the recipient may feel under obligation to reciprocate the received gift, which may burden them. Furthermore, where the donor has specific expectations of the recipient and their lifestyle, this may strain the relationship. It should be noted that these studies are based on interviews, mostly retrospective, with an inherent risk of reporting bias, as donors who have had a negative experience may be more reluctant to share this. There is thus a need for prospective studies of donor parents and their recipient children with longer-term assessment of physical and psychological well-being following donation. It is important for transplant units to enable parents to make a free decision about kidney donation, with no sense of obligation, and also to provide psychological support for them throughout the process. Maintaining realistic expectations also impacts significantly on perceived outcomes.

## Special considerations

### Donors with kidney stones

Incidental kidney stones are found in between 4 and 15% of potential kidney donors [36, 37] and it is important to understand the risk that this confers on both donor and recipient. Mayo Clinic reported one of the largest series in which, among 1957 kidney donors, 53 (2.7%) reported previous symptomatic kidney stones and 210 (11%) had computed tomography (CT) evidence of current kidney stones [38]. Interestingly, in this cohort, the presence of asymptomatic stones was not associated with common risk factors seen in patients with symptomatic stone disease, such as metabolic syndrome, obesity, hypertension and male gender.

In a retrospective analysis of 377 CT angiograms of potential kidney donors between October 2004 and May 2007, Olsburgh et al. found asymptomatic kidney stones in 55 (5%) patients, of whom 20 donated their kidney, and 17 underwent ex vivo ureteroscopy (stone size 2–12 mm, non-obstructing). After a mean follow-up of 10 months, there was no stone-related complication in the recipient and no stone recurrence in the donors [39]. A similar US study of 325 potential kidney donors between 2000 and 2005 found incidental non-obstructing kidney stones in 24 (7.4%) [40]. The median stone size was 2 mm (range 1–9 mm). Sixteen patients proceeded to donation, 11 donating the stone-containing kidney and 5 the stone-free kidney. Only one recipient developed symptomatic kidney stone disease and was found to have hyperoxaluria. No other recipient and no donor had any complications related to kidney stones after a year of follow-up. It is important to note that in both of the above studies, the donors had no evidence of a metabolic stone-forming tendency.

To investigate whether kidney donors have a higher incidence of kidney stone formation, or require more frequent surgical intervention if they do develop a stone, Thomas et al. assessed a cohort of 2019 Canadian patients who had donated a kidney between 1992 and 2009 and compared them with a control group of 20,190 healthy non-donors [41]. Reassuringly, after a median follow-up of 8.4 years, they saw no significant difference in the rate of kidney stones requiring surgical intervention or hospital attendance for kidney stones between the two groups.

Clinical practice guidelines from the British Transplantation Society and the American Transplant Society suggest that kidney donors with small asymptomatic stones and without metabolic disease may be considered for kidney donation [42, 43]. Appropriate donor and recipient counselling and availability of follow-up are important considerations.

### Obese donors

Worldwide, the proportion of adults with a BMI of ≥ 25 kg/m^2^ rose between 1980 and 2013 from 29 to 37% in men and from 30 to 38% in women [44]. This increased rate of obesity in the general population is inevitably mirrored in the kidney donor population. The risk of hypertension, diabetes, metabolic syndrome, ESKD, morbidity and mortality are all increased in overweight and obese individuals [45–47]. Currently, there is significant centre variation in the BMI cut-off for living kidney donation. It is therefore important to know whether obesity adversely affects kidney donors.

From the surgical perspective, there have been concerns about increased complications in overweight and obese donors. Heimbach et al. reported on 553 kidney donors who underwent laparoscopic donor nephrectomy between 1999 and 2003 [48]. A total of 114 had a BMI between 30 and 34.9 kg/m^2^ and 58 had a BMI ≥ 35 kg/m^2^. They found no significant difference in the rate of conversion from laparoscopic to open nephrectomy between the two groups. Operating time was slightly longer in the high BMI group. Wound complications such as infections and seromas were more common in obese (9–10%) compared with non-obese donors (2–4%). Overall peri-operative risk was not significantly higher in the obese group compared with non-obese donors.

Unger et al. reported short-term outcomes following donor nephrectomy in obese versus non-obese donors [49]. In a retrospective analysis of 289 Austrian kidney donors who underwent nephrectomy between 2006 and 2015, 126 donors had a BMI ≤ 25 kg/m^2^, 120 had a BMI between 25 and 30 kg/m^2^ and 43 had a BMI ≥ 30 kg/m^2^. They observed no significant difference between the groups in the conversion rate from laparoscopic to open surgery or in the rate of post-operative complications, such as wound or systemic infection. They noted, however, that both male sex and higher BMI were associated with a statistically significant short-term decline in eGFR during the first post-operative week. The longer-term significance of this observation remains unclear.

In a single centre study of 3752 living kidney donors who underwent nephrectomy between 1975 and 2014, 656 (17.5%) were obese with a BMI ≥ 30 kg/m^2^ [50]. Obese donors were more likely to be older, to be black and to have a higher eGFR at donation. There was no significant difference in intra- or post-operative complications between the obese and non-obese donors, but operating time and length of hospital stay were longer in the obese group. Reassuringly, there was no significant difference in eGFR and risk of ESKD between the two groups after 20 years (*p* = 0.71). Diabetes and hypertension were more common (for diabetes: aHR 3.14; *p* < 0.001 and for hypertension: aHR 1.75; *p* < 0.001) and occurred earlier in obese donors (diabetes mellitus: 12 vs. 18 years post nephrectomy; hypertension: 11 vs. 15 years).

Bellini et al., in a single centre UK study, reported the mean eGFR at 6 months and up to 60 months in 889 living kidney donors between 2000 and 2017. Twenty-six percent of the donors had a BMI ≥ 30 kg/m^2^ pre-donation. The authors found no significant difference in eGFR pre- or post-donation between obese and non-obese donors [51]. This is similar to the findings of Rees et al. who studied 5304 donors, of whom 2108 (40%) were overweight (BMI 25–30 kg/m^2^), 944 (17.8%) were obese (BMI 30–35 kg/m^2^) and 250 (4.7%) were very obese (BMI ≥ 35 kg/m^2^). There was no difference in re-admission or reoperation rate across the BMI groups and no significant difference between BMI groups with respect to decline in eGFR at 6 months [52]. Although the donors with higher BMI had higher blood pressure pre-donation and at 6 months post-donation, the change in blood pressure in each BMI group was similar. Holscher et al. have studied the risk of developing hypertension or diabetes after kidney donation in 41,260 donors who underwent nephrectomy between 2008 and 2014 [53]. A total of 74, 162 and 310 cases per 10,000 developed hypertension at 6 months, 1 and 2 years, respectively. The incidence of hypertension was higher in donors with a higher BMI (relative risk 1.29 per 5 units, 95%CI, 1.17–1.43). Very few donors developed diabetes including 2, 6 and 15 cases per 10,000 donors after 6, 12 and 24 months. The risk of developing diabetes was higher in donors with higher BMI (adjusted incidence rate ratio 1.52 per 5 units; 95%CI, 1.04–2.21). The low incidence of diabetes is most likely related to the short follow-up time. These results again highlight the importance of long-term follow-up for kidney donors.

In a very large study of 119,769 living kidney donors in the US with a maximum of 20-year follow-up, Locke et al. studied the association between BMI and development of ESKD [54]. After adjustment for age, sex, ethnicity, blood pressure and baseline GFR, they found the incidence of ESKD to be 1.9 times higher in obese donors (BMI ≥ 30) compared with non-obese donors (BMI ≤ 30). At 20 years post-donation, the estimated incidence of ESKD was 93.9 per 10,000 in the obese group and 39.7 per 10,000 in the non-obese kidney donors. Obese donors were more likely to be male, black and to have higher blood pressure. Interestingly, they noted a 7% increase in the risk of ESKD for each unit increase in BMI above 27 kg/m^2^, although below this threshold, there was no significant increase in risk.

More recently, the same group has reported the risk of mortality in the same kidney donor population [55]. They found the estimated risk of mortality in obese donors to be 304 per 10,000 and 209 per 10,000 in non-obese living kidney donors. After adjustment for variables such as age, sex, race, eGFR, blood pressure and smoking, the obese kidney donors had a 30% increased risk of long-term mortality compared with the non-obese group (aHR 1.32, 95% CI: 1.09–1.60, *p* = 0.006). It is likely that this increased risk of ESKD and mortality is related to obesity rather than to kidney donation per se.

Most transplant guidelines support kidney donation in otherwise healthy donors with a BMI below 30 kg/m^2^. Donors with a BMI between 30 and 35 kg/m^2^ must be counselled regarding their long-term risk of cardiovascular and kidney disease and should be encouraged to lose weight. As long-term safety data is not available for donors with a BMI greater than 35 kg/m^2^, donation in this population is discouraged.

### Donors of African ancestry

Although donors of African descent represent a minority in most studies of kidney donation, this subgroup frequently demonstrates a higher risk of adverse outcomes, both peri-operatively [56] and in the longer term.

Using data linkage between the Organ Procurement and Transplantation Network (OTPN) between 1987 and 2007 and billing claims from a private health insurer between 2000 and 2007, Lentine et al. [57] investigated the rates of hypertension and kidney disease in 4650 living kidney donors, of whom 13.1% were African American. In comparison with white donors, black donors had an increased risk of hypertension (aHR 1.52; 95%CI, 1.23–1.88) and chronic kidney disease (aHR 2.32; 95% CI, 1.48–3.62). Using data from the same study [56], Lentine et al. also noted that 7 years after donation and after adjustment for age and gender, the African American donors had a higher incidence of kidney-related conditions, including CKD (12.6% vs. 5.6%; aHR 2.32, 95% CI 1.48–3.62), proteinuria (5.7% vs. 2.6%; aHR 2.27, 95% CI 1.32–3.89), nephrotic syndrome (1.3% vs. 0.1%; aHR 15.7, 95% CI 2.97–83.0) and any renal diagnosis (14.9% vs. 9%; aHR 1.71, 95% CI 1.23–2.41).

In the previously mentioned study [13], Muzaale compared the incidence of ESKD in 96,217 donors with 20,024 matched healthy non-donor controls and noted that after an average follow-up of 7.6 years, African American donors had a higher risk of ESKD in comparison with white and Hispanic donors, and also with black healthy controls. At 15 years after donation, the absolute risk of ESKD in black donors was 74.7 per 10,000 versus 23.9 per 10,000 in black non-donors (*p* < 0.001). The absolute risk increase was 50.8 per 10,000 in African-Americans, 25.9 per 10,000 in Hispanics and 22.7 per 10,000 in white donors.

Although some of this increased risk may be attributable to the increased prevalence of hypertension as well as to socioeconomic factors, there is increasing evidence that variants in the gene encoding apolipoprotein L1 (APOL1) might play a contributory role.

Two common protein-changing alleles in the *APOL1* gene are associated with the observed increased risk of ESKD in African Americans [57]. Given that the majority of donors donate to family members [58], it has been proposed that the presence of these genetic variants may alter the course of post-donation kidney function in black donors. To address this, Doshi et al. [59] examined the effect of *APOL1* risk alleles on outcomes in black living kidney donors. Among 136 black donors, 19 (14%) carried two risk alleles (high-risk group) and the remaining 117 carried one or zero risk alleles (low-risk group). The high-risk group had lower pre-donation and post-donation eGFR and at a median of 12-year follow-up showed a more rapid decline in kidney function. Two donors in the high-risk group developed ESKD. They also matched 115 black donors with 115 black non-donor controls; interestingly, the rate of decline in eGFR was similar in both groups irrespective of *APOL1* genotype. Thus, live kidney donation per se does not amplify the risk associated with *APOL1*-related kidney disease. There was no difference in the prevalence of hypertension between donors on the basis of the *APOL1* genotype, although the donors were more likely to develop hypertension than non-donors, even after matching for family history of ESKD and *APOL1* genotype.

Currently, there is insufficient evidence to recommend screening all black donors for the presence of the high-risk *APOL1* genotype. However, the NIH-funded APOL1 Long-term Kidney Transplantation Outcomes Network (APOLLO) [60], which prospectively assesses the effects of *APOL1* variants on kidney outcomes for black living donors and the recipients of their kidneys, could potentially determine the risk of kidney donation in individuals carrying the high-risk gene.

### Future pregnancy

Over half of living kidney donors are female and a significant proportion are of child-bearing age. Given that kidney donation and pregnancy both lead to kidney hyperfiltration, there have been concerns that kidney donation may lead to hypertension during pregnancy with or without pre-eclampsia. A large Norwegian study [61] analysed registry data regarding kidney donors between 1967 and 2002 and identified 726 pregnancies in 326 donors. Of these, 106 pregnancies in 69 donors occurred after donation. The incidence of gestational hypertension and pre-eclampsia was compared between this group and a random sample of 21,000 pregnancies from the medical birth registry. In adjusted analysis, the risk of pre-eclampsia was higher in pregnancies post-donation than in pregnancies pre-donation (5.7% vs. 2.6%, *p* = 0.026). There was no difference between the groups in foetal outcomes such as low birth weight or preterm delivery. A different study by Ibrahim et al. [62] analysed data from 3213 pregnancies in 1085 US donors and compared the incidence of pre-eclampsia pre- and post-donation. They noted an increased risk of gestational hypertension (5.7% vs. 0.6%, *p* < 0.0001), proteinuria (4.3% vs. 1.1%, *p* < 0.0001) and pre-eclampsia (5.5% vs. 0.8%, *p* < 0.0001) in pregnancies after donation. They also found a lower likelihood of full-term delivery in post-donation pregnancies (73.7% vs. 84.6%, *p* = 0.0004) and a higher likelihood of foetal loss (19.2% vs. 11.3%, *p* < 0.0001). However, foetal and maternal outcomes after donation remained comparable to the general population.

In a more recent study [63], Garg et al. retrospectively assessed the incidence of gestational hypertension and pre-eclampsia in Canadian kidney donors between 1992 and 2009. Over a mean follow-up period of 11 years (maximum 20 years), 131 pregnancies in 85 donors were compared with 788 pregnancies in 510 healthy non-donors. After matching for several criteria, they noted that gestational hypertension and pre-eclampsia were more common in kidney donors than in non-donors (11% vs. 5%; odds ratio 2.4; *p* = 0.01). Reassuringly, the incidence of low birth weight and preterm birth was the same in both groups.

It is important to note that in the above studies, donors were slim (BMI < 30) and normotensive. With increasing acceptance of donors with higher BMI and treated hypertension, together with increasing maternal age, it is important to continue surveillance of pregnancy outcomes in past donors. Although the absolute risk of pre-eclampsia remains low, young female donors must be informed of the increased risk.

### Older and younger donors

The increasing need for kidney transplantation coupled with growing confidence regarding the superior outcomes from living kidney transplantation has resulted in increasing uptake in kidney donation from older living donors. Hence, the safety and efficacy of such donations has come under scrutiny. There is no agreed threshold for what constitutes an older donor and different studies have used different thresholds to define older living kidney donors.

To assess the risk of cardiovascular disease and mortality in older donors, Reese et al. [64] matched 3368 individuals older than 55 years who had donated a kidney between 1996 and 2006 with the same number of healthy non-donors selected from a health and retirement study database. The mean age in both groups was 59 years. After a median follow-up of 7.8 years, they noted no significant difference in mortality (4.9 vs. 5.6 deaths per 1000 person-years, HR 0.90, 95%CI 0.71–1.15, *p* = 0.21) or in a composite cardiovascular outcome including ischaemic heart disease, congestive heart failure, stroke, peripheral vascular disease or death (HR 1.02, 95%CI 0.87–1.20, *p* = 0.7).

Another study of 219 healthy US adult kidney donors older than 70 years [65] showed that mortality in this group was lower than in healthy matched controls from the National Health and Nutrition Examination Survey (HR 0.37, 95%CI 0.21–0.65, *p* < 0.001). This was perhaps due to stringent donor selection criteria in this age group. However, graft loss from older donors was noted to be higher than that from younger living donors aged between 50 and 59 (subhazard ratio (SHR) 1.62, 95%CI 1.16–2.28, *p* = 0.005) and closer to outcomes for kidney transplants from matched 50–59-year-old deceased donors (SHR 1.19, 95%CI 0.87–1.63, *p* = 0.3).

Given that older living donors have a lower pre-donation GFR, the inevitable reduction in GFR post-donation is another important concern. Dols et al. [66] have assessed long-term kidney function in 539 living kidney donors who donated between 1994 and 2006. A total of 422 of this group were less than 60 years of age and 117 were more than 60 years old. The older group had a lower pre-donation GFR (80 vs. 96 ml/min, *p* < 0.001). After a median of 5.5 years of follow-up, the rate of GFR decline was similar in both groups (38 ± 9%). At 5 years after donation, more of the older donors had a GFR below 60 ml/min (80% vs. 31%, *p* < 0.001) but no donor had a GFR below 30 ml/min. Interestingly, in this study, recipient outcome did not differ between older and younger donors. Given the shorter life expectancy of older donors, it may be reasonable to accept a lower post-donation eGFR in this group. This is reflected in the GFR thresholds for living kidney donation in the British Transplantation Society Clinical Practice Guidelines [42].

In the previously cited US registry study by Muzaale et al. [13], although the cumulative incidence of ESKD per 10,000 was low among living kidney donors overall, there was significant variation with donor age, being 29.4 in donors aged between 18 and 39, 17.4 in donors aged between 40 and 49, 54.6 in those aged between 50 to 59 and 70.2 in those older than 60 years.

There is also heightened concern about accepting very young individuals as kidney donors [16]. Younger people have a long length of life ahead of them, and, as life expectancy increases and lifestyles change, it is increasingly difficult to make accurate predictions about the degree and nature of risk that a young person may encounter during their lifetime. Long-term prospective studies will improve our understanding about these risks, but in the meantime, it is important to communicate this lack of certainty to younger potential donors.

## Conclusion

Living donor kidney transplantation is widely regarded as the gold standard treatment for patients with ESKD. Yet, clinicians have an obligation to accurately inform potential donors about short- and long-term health concerns that may arise as a consequence of kidney donation and to protect them from excessive risk (Table 1). Accurate estimation of donor risk is challenging, partly because kidney failure and other complications such as hypertension and cardiovascular disease often manifest slowly, so very long follow-up of large and demographically diverse donor cohorts is necessary. Furthermore, kidney donors are generally fitter and healthier than the general public and have undergone extensive health screening, so for a study to be meaningful appropriate controls must be identified.

**Table 1:** Summary of the risks of living kidney donation

Nature of risk	Summary of current evidence	References
Perioperative risk	Perioperative risk relatively low: major complications around 2.5%; overall risk between 7 and 17%, mainly bleeding and infection. Increased risk conferred by black ethnicity, obesity, haematological disorders, psychiatric conditions and robotic nephrectomy.	[4, 5]
Mortality	Short-term (90 day) mortality risk: 3.1 in 10,000. Long-term mortality equivalent to control populations; one study reports higher mortality after very long-term (25-year) follow-up.	[5, 6, 9–12]
End-stage kidney disease	Overall prevalence low, but 3–5 times higher than in well-matched controls. Increased risk conferred by younger age, black ethnicity, male gender, lower GFR, smoking history and the presence of hypertension, albuminuria, obesity or diabetes.	[11, 13, 14]
Hypertension and cardiovascular risk	Some studies report a 2–4-fold increased risk of hypertension. Increased risk conferred by black ethnicity. No significantly increased risk of cardiovascular events.	[9, 18–22]
Gout	Slightly increased risk of gout post-donation but similar to the general population. Increased risk conferred by male gender and black ethnicity.	[24–26]
Metabolic bone disease	Observed differences in PTH, vitamin D3 and FGF-23 but no difference in pathological events.	[9, 22, 24, 29]
Psychological outcomes	Reduced HRQoL in the first 3 months, which improves by 12 months after donation. Reduced HRQoL associated with obesity, history of psychiatric difficulty and non-white race. Education, older age and first-degree relation to the recipient are protective factors.	[30–35]
Kidney stone	Donors with no metabolic abnormality and small kidney stones may safely donate.	[36–41]
Obesity	Slightly increased risk of infection and operative time. Increased incidence of hypertension and diabetes. Possible longer-term increased risk of ESKD and mortality.	[45–55]
Black race	Higher relative risk of hypertension, CKD and ESKD. Increased risk of gout. Increased perioperative risk.	[13, 56, 57, 59]
Pregnancy	2.4-fold higher risk of gestational hypertension and pre-eclampsia. No adverse effect on foetal or maternal outcomes.	[61–63]
Older donors	No significant difference in mortality, cardiovascular events or fall in GFR for older donors.	[13, 64–66]

*PTH* parathyroid hormone, *FGF-23* fibroblast growth factor-23, *HRQoL* health-related quality of life, *CKD* chronic kidney disease, *ESKD* end-stage kidney disease, *GFR* glomerular filtration rate

Importantly, the landscape of donation has changed in recent years so that donors who are older, those with higher BMI and those with controlled hypertension are increasingly likely to be accepted for donation, and this may well impact upon long-term donor outcomes. It is important to acknowledge that there is uncertainty about longer-term consequences of living kidney donation in higher-risk donors and to discuss this openly with donor candidates. Furthermore, the risk of donation must be balanced against the benefit for the potential recipient.

Finally, improvements in technology, wider availability and lower cost of investigations such as genotyping and increasing use of digital health records now allow prospective collection of donor data of increasing granularity. This will enable more accurate risk assessment that can be tailored to the individual donor and facilitate more informative pre-donation discussion and counselling.

## References

[1] Tarantino A (2000). Why should we implement living donation in renal transplantation?. Clin Nephrol.

[2] Guild WR, Harrison JH, Merrill JP, Murray J (1955). Successful homotransplantation of the kidney in an identical twin. Trans Am Clin Climatol Assoc.

[3] NHS Blood and Transplant. Living donor kidney transplantation 2020: A UK strategy 2014. www.odt.nhs.uk/pdf/ldkt_2020_strategy.pdf. Accessed May 2019

[4] Lentine KL, Lam NN, Axelrod D, Schnitzler MA, Garg AX, Xiao H, Dzebisashvili N, Schold JD, Brennan DC, Randall H, King EA, Segev DL (2016). Perioperative complications after living kidney donation: a National Study. Am J Transplant.

[5] Kortram K, Ijzermans JN, Dor FJ (2016). Perioperative events and complications in minimally invasive live donor nephrectomy: a systematic review and meta-analysis. Transplantation.

[6] Segev DL, Muzaale AD, Caffo BS, Mehta SH, Singer AL, Taranto SE, McBride MA, Montgomery RA (2010). Perioperative mortality and long-term survival following live kidney donation. JAMA.

[7] Steiner CA, Bass EB, Talamini MA, Pitt HA, Steinberg EP (1994). Surgical rates and operative mortality for open and laparoscopic cholecystectomy in Maryland. N Engl J Med.

[8] Birkmeyer JD, Siewers AE, Finlayson EV, Stukel TA, Lucas FL, Batista I, Welch HG, Wennberg DE (2002). Hospital volume and surgical mortality in the United States. N Engl J Med.

[9] Garg AX, Meirambayeva A, Huang A, Kim J, Prasad GV, Knoll G, Boudville N, Lok C, McFarlane P, Karpinski M, Storsley L, Klarenbach S, Lam N, Thomas SM, Dipchand C, Reese P, Doshi M, Gibney E, Taub K, Young A, Donor Nephrectomy Outcomes Research Network (2012). Cardiovascular disease in kidney donors: matched cohort study. BMJ.

[10] Okamoto M, Akioka K, Nobori S, Ushigome H, Kozaki K, Kaihara S, Yoshimura N (2009). Short- and long-term donor outcomes after kidney donation: analysis of 601 cases over a 35-year period at Japanese single center. Transplantation.

[11] Mjøen G, Hallan S, Hartmann A, Foss A, Midtvedt K, Øyen O, Reisæter A, Pfeffer P, Jenssen T, Leivestad T, Line PD, Øvrehus M, Dale DO, Pihlstrøm H, Holme I, Dekker FW, Holdaas H (2014). Long-term risks for kidney donors. Kidney Int.

[12] O'Keeffe LM, Ramond A, Oliver-Williams C, Willeit P, Paige E, Trotter P, Evans J, Wadström J, Nicholson M, Collett D, Di Angelantonio E (2018). Mid- and long-term health risks in living kidney donors: a systematic review and meta-analysis. Ann Intern Med.

[13] Muzaale AD, Massie AB, Wang MC, Montgomery RA, McBride MA, Wainright JL, Segev DL (2014). Risk of end-stage renal disease following live kidney donation. JAMA.

[14] Grams ME, Sang Y, Levey AS, Matsushita K, Ballew S, Chang AR, Chow EK, Kasiske BL, Kovesdy CP, Nadkarni GN, Shalev V, Segev DL, Coresh J, Lentine KL, Garg AX; Chronic Kidney Disease Prognosis Consortium (2016). Kidney-failure risk projection for the living kidney-donor candidate. N Engl J Med.

[15] Matas AJ, Berglund DM, Vock DM, Ibrahim HN (2018). Causes and timing of end-stage renal disease after living kidney donation. Am J Transplant.

[16] Steiner RW (2019) “You can't get there from here”: Critical obstacles to current estimates of the ESRD risks of young living kidney donors. Am J Transplant 19:32–36 10.1111/ajt.15089 30137698

[17] Hoy WE, Hughson MD, Bertram JF, Douglas-Denton R, Amann K (2005). Nephron number, hypertension, renal disease, and renal failure. J Am Soc Nephrol.

[18] Sanchez OA, Ferrara LK, Rein S, Berglund D, Matas AJ, Ibrahim HN (2018). Hypertension after kidney donation: incidence, predictors, and correlates. Am J Transplant.

[19] Thiel GT, Nolte C, Tsinalis D, Steiger J, Bachmann LM (2016). Investigating kidney donation as a risk factor for hypertension and microalbuminuria: findings from the Swiss prospective follow-up of living kidney donors. BMJ Open.

[20] Kasiske BL, Anderson-Haag T, Israni AK, Kalil RS, Kimmel PL, Kraus ES, Kumar R, Posselt AA, Pesavento TE, Rabb H, Steffes MW, Snyder JJ, Weir MR (2015). A prospective controlled study of living kidney donors: three-year follow-up. Am J Kidney Dis.

[21] Doshi MD, Goggins MO, Li L, Garg AX (2013). Medical outcomes in African American live kidney donors: a matched cohort study. Am J Transplant.

[22] Moody WE, Ferro CJ, Edwards NC, Chue CD, Lin EL, Taylor RJ, Cockwell P, Steeds RP, Townend JN, CRIB-Donor Study Investigators (2016). Cardiovascular effects of unilateral nephrectomy in living kidney donors. Hypertension.

[23] Krishnan E (2012). Reduced glomerular function and prevalence of gout: NHANES 2009-10. PLoS One.

[24] Kasiske BL, Anderson-Haag T, Ibrahim HN, Pesavento TE, Weir MR, Nogueira JM, Cosio FG, Kraus ES, Rabb HH, Kalil RS, Posselt AA, Kimmel PL, Steffes MW (2013). A prospective controlled study of kidney donors: baseline and 6-month follow-up. Am J Kidney Dis.

[25] Lam NN, McArthur E, Kim SJ, Prasad GV, Lentine KL, Reese PP, Kasiske BL, Lok CE, Feldman LS, Garg AX, Donor Nephrectomy Outcomes Research (DONOR) Network (2015). Gout after living kidney donation: a matched cohort study. Am J Kidney Dis.

[26] Lam NN, Garg AX, Segev DL, Schnitzler MA, Xiao H, Axelrod D, Brennan DC, Kasiske BL, Tuttle-Newhall JE, Lentine KL (2015). Gout after living kidney donation: correlations with demographic traits and renal complications. Am J Nephrol.

[27] Singh JA (2013). Racial and gender disparities among patients with gout. Curr Rheumatol Rep.

[28] Maynard JW, McAdams-DeMarco MA, Law A, Kao L, Gelber AC, Coresh J, Baer AN (2014). Racial differences in gout incidence in a population-based cohort: atherosclerosis risk in communities study. Am J Epidemiol.

[29] Young A, Hodsman AB, Boudville N, Geddes C, Gill J, Goltzman D, Jassal SV, Klarenbach S, Knoll G, Muirhead N, Prasad GV, Treleaven D, Garg AX, Donor Nephrectomy Outcomes Research (DONOR) Network (2012). Bone and mineral metabolism and fibroblast growth factor 23 levels after kidney donation. Am J Kidney Dis.

[30] Wirken L, van Middendorp H, Hooghof CW, Rovers MM, Hoitsma AJ, Hilbrands LB, Evers AW (2015). The course and predictors of health-related quality of life in living kidney donors: a systematic review and meta-analysis. Am J Transplant.

[31] Gross CR, Messersmith EE, Hong BA, Jowsey SG, Jacobs C, Gillespie BW, Taler SJ, Matas AJ, Leichtman A, Merion RM, Ibrahim HN, RELIVE Study Group (2013). Health-related quality of life in kidney donors from the last five decades: results from the RELIVE study. Am J Transplant.

[32] Kärrfelt HM, Berg UB, Lindblad FI, Tydén GE (1998). To be or not to be a living donor: questionnaire to parents of children who have undergone renal transplantation. Transplantation.

[33] Kärrfelt HM, Berg UB, Lindblad FI (2000). Renal transplantation in children: psychological and donation-related aspects from the parental perspective. Pediatr Transplant.

[34] Thys K, Schwering KL, Siebelink M, Dobbels F, Borry P, Schotsmans P, Aujoulat I, ELPAT Pediatric Organ Donation and Transplantation Working Group (2015). Psychosocial impact of pediatric living-donor kidney and liver transplantation on recipients, donors, and the family: a systematic review. Transpl Int.

[35] Buer LC, Hofmann BM (2012). How does kidney transplantation affect the relationship between donor and recipient?. Tidsskr Nor Laegeforen.

[36] Scales CD, Smith AC, Hanley JM, Saigal CS, Project UdiA (2012). Prevalence of kidney stones in the United States. Eur Urol.

[37] Romero V, Akpinar H, Assimos DG (2010). Kidney stones: a global picture of prevalence, incidence, and associated risk factors. Rev Urol.

[38] Lorenz EC, Lieske JC, Vrtiska TJ (2011). Clinical characteristics of potential kidney donors with asymptomatic kidney stones. Nephrol Dial Transplant.

[39] Olsburgh J, Thomas K, Wong K, Bultitude M, Glass J, Rottenberg G, Silas L, Hilton R, Koffman G (2013). Incidental renal stones in potential live kidney donors: prevalence, assessment and donation, including role of ex vivo ureteroscopy. BJU Int.

[40] Kim IK, Tan JC, Lapasia J, Elihu A, Busque S, Melcher ML (2012). Incidental kidney stones: a single center experience with kidney donor selection. Clin Transpl.

[41] Thomas SM, Lam NN, Welk BK, Nguan C, Huang A, Nash DM, Prasad GV, Knoll GA, Koval JJ, Lentine KL, Kim SJ, Lok CE, Garg AX, Donor Nephrectomy Outcomes Research (DONOR) Network (2013). Risk of kidney stones with surgical intervention in living kidney donors. Am J Transplant.

[42] Guidelines for Living Donor Kidney Transplantation (2018) https://bts.org.uk/wp-content/uploads/2018/07/FINAL_LDKT-guidelines_June-2018.pdf Accessed May 2019

[43] American Society of Transplantation. Kidney donation for people with kidney stones. Live Donor Toolkit: Resources for Those Considering Live Donation. http://www.livedonortoolkit.com/medical-toolkit/kidney-donation-people-kidney-stones Accessed May 2019

[44] Ng M, Fleming T, Robinson M (2014). Global, regional, and national prevalence of overweight and obesity in children and adults during 1980-2013: a systematic analysis for the global burden of disease study 2013. Lancet.

[45] Whitlock G, Lewington S, Sherliker P, Clarke R, Emberson J, Halsey J, Qizilbash N, Collins R, Peto R, Prospective Studies Collaboration (2009). Body-mass index and cause-specific mortality in 900,000 adults: collaborative analyses of 57 prospective studies. Lancet.

[46] Hsu CY, McCulloch CE, Iribarren C, Darbinian J, Go AS (2006). Body mass index and risk for end-stage renal disease. Ann Intern Med.

[47] Nguyen NT, Magno CP, Lane KT, Hinojosa MW, Lane JS (2008). Association of hypertension, diabetes, dyslipidemia, and metabolic syndrome with obesity: findings from the National Health and nutrition examination survey, 1999 to 2004. J Am Coll Surg.

[48] Heimbach JK, Taler SJ, Prieto M, Cosio FG, Textor SC, Kudva YC, Chow GK, Ishitani MB, Larson TS, Stegall MD (2005). Obesity in living kidney donors: clinical characteristics and outcomes in the era of laparoscopic donor nephrectomy. Am J Transplant.

[49] Unger LW, Feka J, Sabler P, Rasoul-Rockenschaub S, Györi G, Hofmann M, Schwarz C, Soliman T, Böhmig G, Kainz A, Salat A, Berlakovich GA (2017). High BMI and male sex as risk factor for increased short-term renal impairment in living kidney donors - retrospective analysis of 289 consecutive cases. Int J Surg.

[50] Serrano OK, Sengupta B, Bangdiwala A, Vock DM, Dunn TB, Finger EB, Pruett TL, Matas AJ, Kandaswamy R (2018). Implications of excess weight on kidney donation: long-term consequences of donor nephrectomy in obese donors. Surgery.

[51] Bellini MI, Charalampidis S, Stratigos I, Dor FJMF, Papalois V (2019). The effect of Donors' demographic characteristics in renal function post-living kidney donation. Analysis of a UK single Centre cohort. J Clin med.

[52] Reese PP, Feldman HI, Asch DA, Thomasson A, Shults J, Bloom RD (2009). Short-term outcomes for obese live kidney donors and their recipients. Transplantation.

[53] Holscher CM, Bae S, Thomas AG, Henderson ML, Haugen CE, DiBrito SR, Muzaale AD, Garonzik Wang JM, Massie AB, Lentine KL, Segev DL (2019). Early hypertension and diabetes after living kidney donation: a National Cohort Study. Transplantation.

[54] Locke JE, Reed RD, Massie A, MacLennan PA, Sawinski D, Kumar V, Mehta S, Mannon RB, Gaston R, Lewis CE, Segev DL (2017). Obesity increases the risk of end-stage renal disease among living kidney donors. Kidney Int.

[55] Locke JE, Reed RD, Massie AB, MacLennan PA, Sawinski D, Kumar V, Snyder JJ, Carter AJ, Shelton BA, Mustian MN, Lewis CE, Segev DL (2019). Obesity and long-term mortality risk among living kidney donors. Surgery.

[56] Lentine KL, Schnitzler MA, Xiao H, Saab G, Salvalaggio PR, Axelrod D, Davis CL, Abbott KC, Brennan DC (2010). Racial variation in medical outcomes among living kidney donors. N Engl J Med.

[57] Lentine KL, Schnitzler MA, Garg AX, Xiao H, Axelrod D, Tuttle-Newhall JE, Brennan DC, Segev DL (2015). Race, relationship and renal diagnoses after living kidney donation. Transplantation.

[58] Dummer PD, Limou S, Rosenberg AZ, Heymann J, Nelson G, Winkler CA, Kopp JB (2015). APOL1 kidney disease risk variants: an evolving landscape. Semin Nephrol.

[59] Doshi MD, Ortigosa-Goggins M, Garg AX, Li L, Poggio ED, Winkler CA, Kopp JB (2018). Genotype and renal function of black living donors. J Am Soc Nephrol.

[60] APOL1 Long-Term Kidney Transplantation Outcomes Network (APOLLO) Clinical Centers: (Collaborative U01), Bethesda, MD, National Institutes of Health. Published 2017

[61] Reisaeter AV, Røislien J, Henriksen T, Irgens LM, Hartmann A (2009). Pregnancy and birth after kidney donation: the Norwegian experience. Am J Transplant.

[62] Ibrahim HN, Akkina SK, Leister E, Gillingham K, Cordner G, Guo H, Bailey R, Rogers T, Matas AJ (2009). Pregnancy outcomes after kidney donation. Am J Transplant.

[63] Garg AX, McArthur E, Lentine KL, Network DNORD (2015). Gestational hypertension and preeclampsia in living kidney donors. N Engl J Med.

[64] Reese PP, Bloom RD, Feldman HI, Rosenbaum P, Wang W, Saynisch P, Tarsi NM, Mukherjee N, Garg AX, Mussell A, Shults J, Even-Shoshan O, Townsend RR, Silber JH (2014). Mortality and cardiovascular disease among older live kidney donors. Am J Transplant.

[65] Berger JC, Muzaale AD, James N, Hoque M, Wang JM, Montgomery RA, Massie AB, Hall EC, Segev DL (2011). Living kidney donors ages 70 and older: recipient and donor outcomes. Clin J Am Soc Nephrol.

[66] Dols LF, Kok NF, Roodnat JI, Tran TC, Terkivatan T, Zuidema WC, Weimar W, Ijzermans JN (2011). Living kidney donors: impact of age on long-term safety. Am J Transplant.

